# Beneficial effect of consuming milk containing only A2 beta-casein on gut microbiota: A single-center, randomized, double-blind, cross-over study

**DOI:** 10.1371/journal.pone.0323016

**Published:** 2025-05-08

**Authors:** Chin-Hee Song, Nayoung Kim, Yonghoon Choi, Seulgi Kim, Kyung Su Kim, Min Hee Park, Sang Hee Lee, Dong Ho Lee

**Affiliations:** 1 Department of Internal Medicine, Seoul National University Bundang Hospital, Seongnam, South Korea; 2 Research Center for Sex- and Gender-Specific Medicine, Seoul National University Bundang Hospital, Seongnam, South Korea; 3 Department of Internal Medicine and Liver Research institute, Seoul National University College of Medicine, Seoul, South Korea; 4 R&D Center, Seoul Dairy Cooperative, Ansan, South Korea; Wageningen Universiteit, NETHERLANDS, KINGDOM OF THE

## Abstract

Cow milk contains essential nutrients, with β-casein existing in A1 and A2 forms. Studies suggest that A2 milk (containing only A2 β-casein) may offer gastrointestinal (GI) benefits compared to A1/A2 milk (containing both forms). This study investigated the effects of A2 milk consumption on the gut microbiota of South Korean cohort experiencing GI discomfort after consuming A1/A2 milk. Thirty-five participants with GI discomfort after milk consumption were included. Stool DNA was analyzed using 16S rRNA gene sequencing before and after consuming either A1/A2 or A2 milk. Beta diversity analysis using the generalized UniFrac distance method revealed a significant shift in gut microbiota composition after A2 milk consumption (*p* = 0.04), but no significant change after consuming A1/A2 milk. Significant differences in gut microbiota composition were found between A1/A2 and A2 milk drinkers after milk consumption (*p* = 0.031). Alpha diversity indices remained unchanged. Notable increases in beneficial microbes, including *Bifidobacterium* and *Blautia*, were observed after A2 milk intake. Linear discriminant analysis Effect Size (LEfSe) analysis identified significant enrichment of Actinobacteria, particularly *Bifidobacterium longum* and *Blautia wexlerae*, in the A2 group. Phylogenetic Investigation of Communities by Reconstruction of Unobserved States (PICRUSt) analysis highlighted enriched transport systems related to energy, peptides, sugars, and raffinose family oligosaccharides in the A2 group. Spearman correlation showed significant associations between *Bifidobacterium*, *Blautia*, and enhanced transport systems exclusively in the A2 group. Two weeks of A2 milk consumption led to significant alterations in gut microbiota, promoting beneficial microbes and related functions. A2 milk could be a suitable alternative for subjects who experience milk-intake-related GI discomfort.

## Introduction

Cow milk is a fundamental component of the human diet, providing essential nutrients that support growth and development across all ages [1]. Casein, which constitutes about 80% of the protein content in cow milk, is categorized into four main types: αs1- (39–46%), αs2- (8–11%), β- (25–35%), and κ-casein (8–15%) [2]. Among these casein types, β-casein exists in multiple forms, with A1 and A2 β-casein being the most common [3]. Regular milk (A1/A2 milk) contains both A1 and A2 β-casein, whereas A2 milk contains A2 β-casein only. Recent studies suggest that A2 milk may offer gastrointestinal (GI) benefits over regular milk, potentially reducing GI discomfort in some individuals.

β-Casomorphin-7 (BCM-7) is released during the digestion of A1 β-casein, and it has been reported to be linked to delayed gastric emptying and increased GI transit time in animal models [4]. As an opioid peptide, BCM-7 interacts with μ-opioid receptors, thereby influencing GI motility, mucus production, and hormone secretion [5]. Additionally, BCM-7 has been suggested to inhibit lactase production, leading to increased colonic inflammation and lactose fermentation owing to increased GI transit time [5]. This interaction may exacerbate the symptoms of lactose intolerance, including diarrhea, abdominal pain, borborygmus, and flatus. Lactose intolerance is prevalent worldwide, particularly among American Indians and Asians [6,7]. In South Korea, a substantial variation of its prevalence has been reported, ranging from 39.1% to 84.1% [8]. This suggests that a significant portion of the population may experience GI discomfort after consuming milk because of lactose intolerance and the A1 β-casein-derived release of BCM-7.

Furthermore, BCM-7 exposure in the GI tract has been shown to increase microbiome stress [9]. BCM-7 may affect the production and activation of lactase, and unabsorbed lactose could contribute to colonic inflammation by altering gut microbiota composition [10]. In contrast to A1 β-casein, recent study using mouse models has reported the beneficial effects of A2 β-casein, showing that A2 β-casein alleviates gut microbiota disorders in immunosuppressed mice by regulating the relative abundance of beneficial bacteria such as *Oscillospira*, *Lactobacillus*, and *Bifidobacteria*, while reducing harmful bacteria such as *Coprococcus* and *Desulfovibrionaceae* [11]. Another study from China found that A1 β-casein consumption leads to reduced short-chain fatty acid (SCFA) levels and suggested a possible association with symptoms [12].

Epidemiological studies have highlighted the role of milk in the prevention of chronic conditions, such as cardiovascular diseases, osteoporosis [13], some cancers, obesity, and diabetes [1]. The anti-inflammatory properties of milk may be beneficial, because its bioactive proteins have been suggested to mitigate intestinal inflammation [14,15]. Extensive research over the past decades has established that the human gut microbiota, a complex ecosystem of trillions of microorganisms, plays a crucial role in maintaining overall health [16]. Many human disorders are associated with gut microbiota, including GI disorders, such as inflammatory bowel disease [17] and colorectal cancer [18], as well as metabolic diseases, such as diabetes mellitus [19] and obesity [20]. Gut microbiota also affects the immune homeostasis of the host [21]. Gut microbial composition can be positively or negatively influenced by various dietary and lifestyle factors [22], and dairy products are known to affect gut microbial diversity and the relative abundance of specific taxa [23–25]. Metataxonomic sequencing of highly conserved 16S ribosomal RNA (rRNA) is commonly used to identify the composition of the gut bacterial community. Analysis tools like PICRUSt could further predict microbial genes within the microbiota based on the Kyoto Encyclopedia of Genes and Genomes (KEGG) orthology, aiding in the understanding of microbial metabolism, such as butyric acid production, which influences host health [26].

Notably, previous human trials in New Zealand, China, Australia, and the United States have shown a correlation between A1 β-casein consumption and decreased GI motility [5], suggesting that milk-intake-related GI discomfort may be attributed to a greater degree to A1 β-casein than lactose intolerance [12]. Recently, our team has reported that drinking A2 milk may have alleviated GI discomfort in a South Korean cohort, while A1/A2 milk consumption did not [27].

Despite extensive research on the effects of fermented dairy products on gut microbiota, there is limited information on how the consumption of regular milk affects the human gut microbiome, particularly in population-level observational studies [28]. In a previous randomized crossover study, we compared the consumption of A2 milk with regular milk in South Koreans who experienced symptoms of GI discomfort following milk consumption [27]. In that earlier study, we observed the beneficial effects of consuming A2 milk, such as an improvement in GI discomfort symptom scores and fecal calprotectin levels that are markers of colonic inflammation [27]. We hypothesized that these effects of A2 milk consumption may be related to its beneficial effects on gut microbiota. Therefore, the aim of the present study was to identify the beneficial gut microorganisms associated with A2 milk consumption by evaluating the differences in gut microbiota composition before and after consumption of A2 milk compared with A1/A2 milk. Additionally, the study sought to analyze the predictive functional profiling of microbial communities, particularly in transport systems, based on KEGG modules.

## Materials and methods

### Milk preparation

The percentage of beta-casein in A1/A2 milk used in the clinical trial was determined through DNA ratio testing using PCR, leading to the selection of two final farms. Upon direct analysis of the raw milk from these two farms, as well as through calculations based on Ct values provided by our contracted agency (Biomed) in conjunction with Seoul Milk, the average ratio of A1 to A2 beta-casein was found to be 40 ± 3:60 ± 3 (mean±SD). And, A2 milk used in this study contains 100% A2 beta-casein (from A2/A2 genetic type bovine).

### Study design and participants

**Fig 1:**
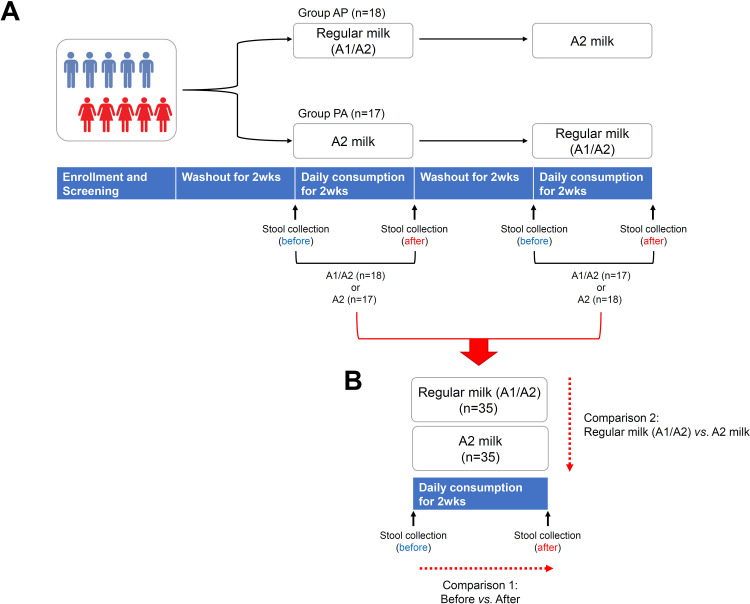
Study design and analysis scheme. (A) Study design. Group 1, A1/A2 milk consumption followed by A2 milk consumption (A1/A2 → A2); Group 2, A2 milk consumption followed by A1/A2 milk consumption (A2 → A1/A2). (B) Analysis scheme. Gut microbiome data obtained from fecal samples of 35 participants were analyzed as follows: first, a comparison before and after consumption of A1/A2 milk and A2 milk; second, a comparison between A1/A2 milk and A2 milk after consumption.

The clinical setting of the present study has been described in our previous report [27]. Briefly, this was a randomized, double-blind, crossover human trial involving participants who experienced GI discomfort after milk consumption. Fifty participants aged > 19 years were recruited between April 7, 2023 and November 20, 2023 [27], while the gut microbiome data analysis, as a secondary outcome of the previous study, was performed between January 6, 2024, and August 16, 2024. The mean age of participants in the AP group was 37.47 ± 13.30 years, while in the PA group, it was 37.11 ± 14.38 years. The proportion of female participants was 29.4% in the AP group and 27.8% in the PA group. There was no difference between PA and AP group in terms of age and sex. Baseline screening tests were conducted 2 weeks before the first scheduled intervention. Eligibility was based on our inclusion and exclusion criteria (S1 Table). Briefly, individuals with severe lactose intolerance, those who have taken antibiotics within two weeks prior to the study, and those who have taken gastrointestinal medications including probiotics within 7 days prior to the study were excluded. As shown in Fig 1A, the registered participants were randomly assigned to one of two groups, and for each intervention period, either A2 milk (A2/A2) or regular milk (A1/A2) was consumed for 2 weeks following a 2-week washout period. Then A1/A2 or A2 milk was consumed for 2 weeks. The efficacy and safety of the intervention were assessed after each 2-week period and have been reported [27].

The daily milk intake was set at 500 mL, providing 25g of lactose per day, based on hydrogen breath test standards and previous clinical studies [29]. To minimize potential errors in sample preparation due to weighing, an additional 10% was included in the total amount. A two-week washout period was implemented before supplementation to minimize residual effects from prior dairy consumption. During this period, participants were instructed to maintain their usual dietary habits, physical activity levels, and overall dietary intake while avoiding dairy products. Dietary compliance was monitored through dietary survey questionnaires at multiple visits. Participants recorded their daily intake of restricted foods, including additional milk, fermented dairy, cheese, cream, and other dairy-based products, which were reviewed at subsequent visits to ensure adherence and minimize potential confounding effects. Dietary survey forms were distributed during Visits 1, 2, 3, and 4, and collected at Visits 2, 3, 4, and 5 to confirm compliance.

Participants were randomly assigned using the permuted block randomized method, and the trial was double-blinded to minimize bias [27]. During the trial, the use of medications that could affect the outcome of the trial were prohibited, and the individual history of milk consumption was checked through questionnaires at each visit. To maintain the double-blind nature of the trial, the details of the information on randomization for each group were sealed and remained undisclosed by the administrator [27]. The study protocol was registered on ClinicalTrials.gov (NCT06252636) and Clinical Research Information Service (CRIS) (KCT0009301) and was approved by the Seoul National University Bundang Hospital Institutional Review Board (IRB) (IRB Approval Number: B-2302-808-001). Written informed consent was obtained from all the participants.

### Fecal sample collection and stool DNA extraction

Fecal samples were collected by participants at home and stored in a cooling bag until their hospital visit. During transportation to the research facility, the fecal samples were transferred by researchers with dry ice to maintain a temperature of -20°C. The collected fecal samples were stored at -80°C until analysis. Fecal total DNA was extracted using the QIAGEN Fast DNA Stool Mini Kit (QIAGEN, USA), following the manufacturer’s instructions. A quantification of extracted fecal total DNA was performed using Quant-iT™ PicoGreen™ dsDNA Assay Kit (Thermo Fisher Scientific, Wilmington, DE, USA).

### Miseq sequencing

For MiSeq sequencing, PCR amplification, amplicon purification, and 16S ribosomal RNA (rRNA) library preparation procedures were conducted as follows:

The PCR mixture contained adjusted total DNA (5 ng/μL), KAPA HiFi HotStart ReadyMix (Roche, Switzerland) and universal primers targeting the V3 to V4 regions of the bacterial 16S rRNA gene (341F: 5’-CCTACGGGNGGCWGCAG-3’, 805R: 5’-GACTACHVGGGTATCTAATCC-3’). The amplification protocol was as follows: an initial denaturation step at 95°C for 3 min, followed by 25 cycles of denaturation at 95°C for 30 s, annealing at 55°C for 30 s, extension at 72°C for 30 s, and a final extension step at 72°C for 5 min. Verification of amplicons was performed via gel electrophoresis on a 1% agarose gel (135 V, run for 20 min) supplemented with a 100 bp + DNA ladder (Invitrogen, USA).

Amplicon purification was conducted using AMPure XP beads (Beckman Coulter, USA) according to the instructions of the manufacturer. Subsequently, a 16S rRNA library was prepared using the Nextera XT Index Kit (#FC-131–1001, Illumina, USA). The PhiX Control Kit v3 (# FC-110–3001, Illumina) was employed as an internal standard to ensure sequencing quality control. A quantification of PCR amplicons was conducted using Quant-iT™ PicoGreen™ dsDNA Assay Kit (Thermo Fisher Scientific, Wilmington, DE, USA). The size and integrity of the PCR products were checked using the 4200 TapeStation System (Agilent, USA).

Finally, the 16S rRNA library prepared earlier underwent sequencing on the MiSeq sequencer (Illumina, USA) with the MiSeq Reagent Kit v3 (# MS-102–3003, Illumina), following the Illumina MiSeq 2x300 bp paired-end sequencing protocol.

### Data processing

All data processing steps were conducted using the EzBioCloud platform (CJ Bioscience, South Korea). Paired-end FASTQ files from the MiSeq sequencer were imported into the EzBioCloud platform. To obtain qualified reads, the imported reads were trimmed using the Trimmomatic version 0.32 software with a parameter of > Q25. Trimmed sequences were then merged using VSEARCH software version 2.13.4. Primer sequences in merged reads were trimmed using the alignment algorithm of Myers-Miller with a similarity cutoff of 0.8. Non-specific amplicons were removed using HMMER software version 3.2.1. Redundant reads were clustered, and unique reads were extracted using the “derep_fulllength” command of VSEARCH.

For taxonomic analysis, qualified reads were aligned to the EzBioCloud 16S rRNA database using the “usearch_global” command of VSEARCH. Chimeric reads were filtered using the UCHIME algorithm (with 97% similarity) against a non-chimeric 16S rRNA database. Reads that did not match at the species level (with 97% similarity) in the EzBioCloud database were compiled, and the “cluster_fast” command was used to perform *de novo* clustering to generate additional operational taxonomic units (OTUs).

### Microbiome analysis

#### Alpha and beta diversity analysis.

To determine the appropriate sequencing depth required for diversity analysis, rarefaction curve analysis was conducted. The rarefaction curves of OTUs were generated using the EzBioCloud platform (CJ Bioscience, South Korea). To avoid bias in the results, the reads were normalized to 16,197 and then an analysis was conducted. Various alpha diversity indices were calculated using OTU information. Using the established sequencing depth, a beta diversity analysis was carried out to detect small changes in gut microbiota composition among samples. The analysis used the generalized UniFrac distance method at the species level for each sample, and the results were visualized using a 2D principal coordinate analysis (PCoA) score plot based on the PC1 and PC2 axes.

#### Taxonomic and functional analysis.

The taxonomic classification was performed using the EzBioCloud database. A taxonomic analysis was conducted to examine the overall taxonomic differences between the A2 and A1/A2 milk consumption groups. The relative abundance of the top 10 taxa and each taxon within each group were visualized using bar plots.

To predict the functional composition of the gut microbiota in A2 and A1/A2 milk consumption groups, PICRUSt analysis was performed in the EzBioCloud database, and the KEGG database was used as a reference for functional prediction.

### Statistical analysis and visualization

For beta-diversity analysis, PERMANOVA was applied using the CJ EzBioCloud platform with 999 permutations to assess differences between groups.

Given the crossover study design, a Generalized Linear Model (GLM) was used to evaluate the effects of Treatment, Period, and Group at the alpha-diversity, phylum, genus, and species levels in R using the lmer4 package. The GLM applied is as follows: Response ~ Treatment * Period + Group. The Response represents the relative abundance of each phylum, genus, species or alpha-diversity value while Treatment refers to the consumption of A1 milk or A2 milk. Period indicates the two experimental periods (Period 1 and Period 2), and Group represents the participant groups (PA and AP).

Additionally, Spearman’s rank correlation assessed associations between calprotectin or PICRUSt results and genus and species levels, with both the rho and *p*-values calculated using cor.test (stats package). A significance level of *p *< 0.05 was considered statistically significant. Visualizations were generated using R software version 4.3.3(R Core Team, 2013).

### Data availability and accession number

The unprocessed 16S rRNA gene datasets that were generated in this study are available from the National Center for Biotechnology Information Sequence Read Archive, USA (Accession number: PRJNA1130054; https://www.ncbi.nlm.nih.gov/sra/PRJNA1130054).

## Results

Previously, a randomized, double-blind, cross-over human trial was performed with 40 subjects who experienced GI discomfort following milk consumption. Either A2 milk (A2 → A1/A2) or A1/A2 milk was first consumed for 2 weeks (A1/A2 → A2) for each intervention period, with a 2-week washout period between each period. As results, A2 milk caused less abdominal pain (*P* = 0.050), fecal urgency (*P *< 0.001) and borborygmus (*P* = 0.007) compared to A1/A2 milk in questionnaire for digestive symptoms. In addition, fecal calprotectin also decreased or less increased after consumption of A2 milk compared to A1/A2 milk (*P* = 0.030), and this change was more pronounced in males (*P* = 0.005) than in females. This study analyzed microbiome data as a secondary outcome of a previous study.

### The effect of A2 milk consumption on gut microbiota diversity

To identify beneficial gut microorganisms following A2 milk consumption, high-throughput 16S rRNA gene sequencing was performed on stool DNA from a total of 35 participants (Fig 1A). The analysis scheme was as follows: first, a comparison before and after consumption of A1/A2 and A2 milk; second, a comparison between A1/A2 and A2 milk after consumption. (Fig 1B).

**Fig 2:**
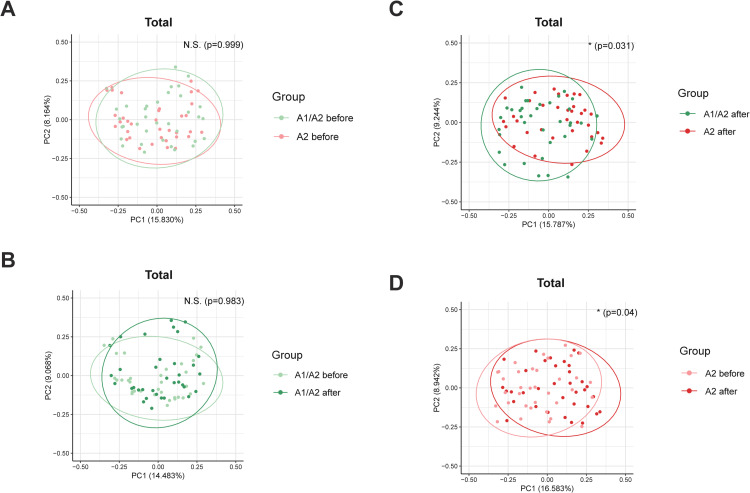
Beta diversity of gut microbiota. Sample clustering by Generalized UniFrac-based PCoA at the species level. (A) PCoA plot comparing samples before and after A1/A2 milk consumption. (B) PCoA plot comparing samples before and after A2 milk consumption. (C) PCoA plot comparing A2 milk and A1/A2 milk before consumption. (D) PCoA plot comparing A2 milk and A1/A2 milk after consumption. Significance for similarity of bacterial population clustering was analyzed by PERMANOVA. *, *p* < 0.05, n.s., no significance. The clustering of each group is marked with a different color: A1/A2 before, yellowish green ellipse; A1/A2 after, green ellipse; A2 before, pink ellipse; A2 after, red ellipse. PCoA, principal coordinates analysis; PERMANOMA, permutational multivariate analysis of variance.

A beta diversity analysis was conducted to assess the differences in gut microbiota composition. PCoA based on the generalized UniFrac distance method was used to visualize the differences in microbial communities. The PCoA plot shows the beta diversity of gut microbiota before and after the consumption of A1/A2 milk. This analysis showed no significant difference in gut microbiota composition before and after A1/A2 milk consumption (Fig 2A). Fig 2B shows a PCoA plot of the beta diversity of gut microbiota before and after A2 milk consumption. This analysis revealed a significant shift in the gut microbiota composition after the consumption of A2 milk (Fig 2B, *p* = 0.04). In Fig 2C, the PCoA plot shows the beta diversity of gut microbiota before the consumption of A1/A2 and A2 milk. There was no significant difference in the gut microbiota composition between these two groups before milk consumption (Fig 2C). In Fig 2D, the PCoA plot depicts the beta diversity of gut microbiota after the consumption of A1/A2 and A2 milk. This analysis indicated a significant difference in the gut microbiota composition between the two groups after milk consumption (Fig 2D, *p* = 0.031). Additionally, we performed a subgroup analysis by dividing the Total group into PA (n = 17) and AP (n = 18), but no significant correlations were observed in either subgroup (S1 Fig). Overall, our beta diversity analysis demonstrated that while the consumption of A1/A2 milk did not significantly alter the gut microbiota composition, the consumption of A2 milk resulted in a significant shift in the gut microbiome. Further, there was a significant difference in the gut microbiota composition between the two groups after milk consumption, highlighting the potential impact of drinking A2 milk on gut microbiota.

**Fig 3:**
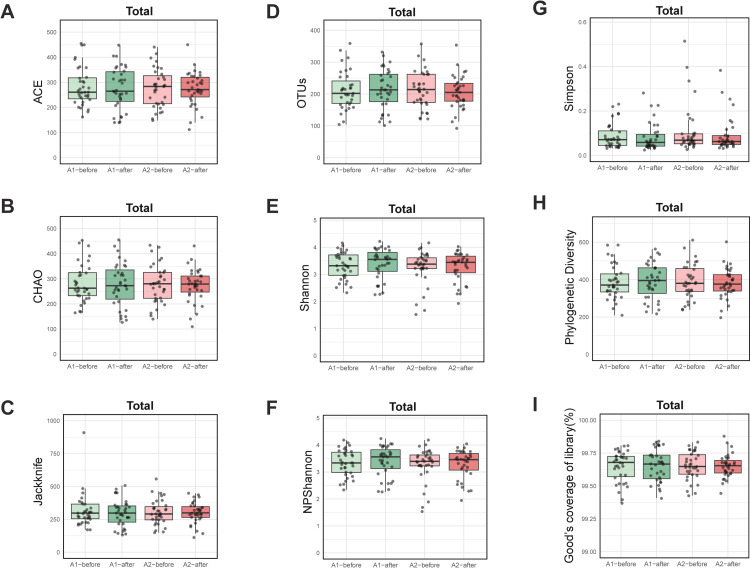
Alpha diversity of gut microbiota. (A) Observed OTU count, (B) ACE, (C) Chao1, (D) Jackknife, (E) Shannon, (F) NPShannon, (G) Simpson, (H) Phylogenetic Diversity, (I) Good’s coverage of library. Statistical analysis for (A-I) was performed using a generalized linear model (GLM). No significant *p*-values for p_treatment (A2 treatment effect), p_period (period effect), and p_group (group effect) were observed, and thus, they are not displayed above the graphs.

We assessed the alpha diversity of gut microbiota using several indices, including OTUs, ACE, Chao1, Jackknife, Shannon, NPShannon, Simpson, Phylogenetic Diversity, and Good’s Coverage in the total group (Fig 3) and its subgroups, PA and AP (S2 Fig). Overall, the alpha diversity indices indicated an absence of any significant changes in gut microbiota diversity between the different groups and conditions tested (Figs 3 and S2). This suggests that neither regular milk nor A2 milk consumption has a substantial impact on the alpha diversity of the gut microbiome in this study.

### Effect of A2 milk consumption on gut microbiota taxonomic composition

**Fig 4:**
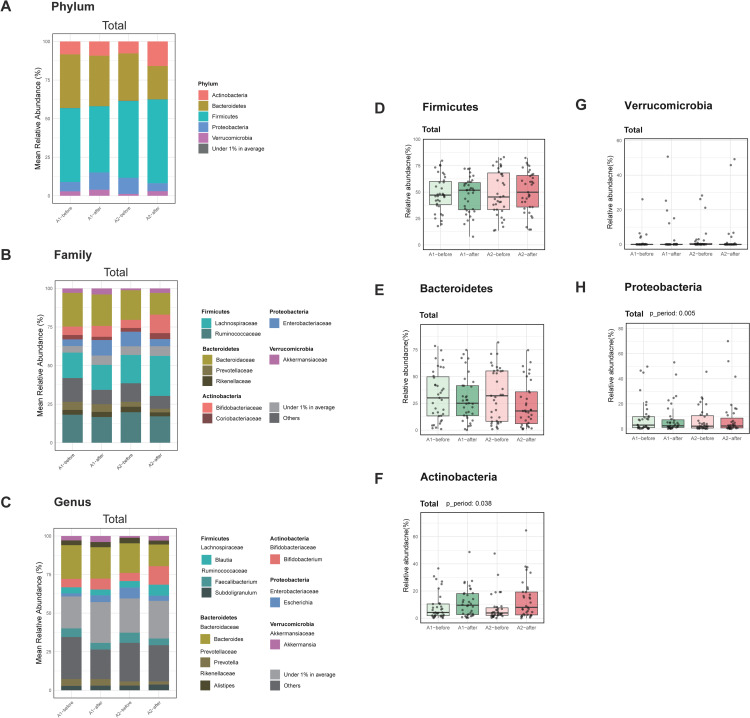
Distribution of gut microbiome before and after consumption of A1/A2 and A2 milk. (A-C) Gut microbiota compositions at the phylum (A), family (B), and genus (C) levels. (D-H) Box plots for relative taxonomic abundance of Firmicutes (D), Bacteroidetes (E), Actinobacteria (F), Verrucomicrobia (G), and Proteobacteria (H). Statistical analysis for (D-H) was performed using a generalized linear model (GLM). Significant *p*-values for p_treatment (A2 treatment effect), p_period (period effect), and p_group (group effect) are displayed above the graphs. Only p_period effects were significant, and these are indicated in the figures.

The relative abundances of major phyla, including Firmicutes, Bacteroidetes, Actinobacteria, Verrucomicrobia, and Proteobacteria (Fig 4A), were analyzed before and after consumption of A1/A2 and A2 milk. Fig 4B and 4C present the taxonomic composition at the phylogenetic family and genus level. The relative abundance of Firmicutes was compared across four milk consumption conditions: A1/A2 before, A1/A2 after, A2 before, and A2 after. There was no significant difference in Firmicutes abundance between A1/A2 before and A1/A2 after, A1/A2 before and A2 before, or A2 before and A2 after (Fig 4D). However, a significant decrease in Firmicutes abundance was observed in A1/A2 after compared to A2 after (Fig 4D, *p* = 0.007). For Bacteroidetes, a significant decrease in relative abundance was noted between A1/A2 after and A2 after (Fig 4E, *p* = 0.034). Comparisons between A1/A2 before and A1/A2 after, A1/A2 before and A2 before, and A2 before and A2 after did not show significant differences (Fig 4E). The analysis revealed a significant increase in Actinobacteria abundance between A1/A2 after and A2 after (*p* = 0.012), and a highly significant increase between A2 before and A2 after (*p* = 0.002) (Fig 4F). There were no significant changes observed between A1/A2 before and A1/A2 after, A1/A2 before and A2 before, or A2 before and A1/A2 after (Fig 4F). No significant differences in Verrucomicrobia abundance were detected between any of the following milk consumption groups (Fig 4G). Similarly, Proteobacteria did not show significant differences across the various milk consumption conditions (Fig 4H). Additionally, we performed a subgroup analysis by dividing the Total group into PA and AP (S3 Fig). In the PA group, the effect of A2 treatment on Actinobacteria was significant (S3F Fig; p_treatment = 0.024), while in the AP group, A2 treatment had a significant effect on Proteobacteria (S3H Fig; p_treatment = 0.029). Overall, our findings indicate specific shifts in the relative abundance of certain gut microbiome taxa following dietary intervention. Notably, significant changes were observed in Firmicutes, Bacteroidetes, and Actinobacteria, suggesting the potential impact of A2 milk consumption on these phyla. The lack of significant changes in Verrucomicrobia and Proteobacteria abundance suggests that these taxa may be less responsive to the milk consumption conditions or more stable in the gut environment. Further studies are needed to elucidate the mechanisms driving these changes and their potential implications for host health.

### Identification of taxonomic biomarkers after A2 milk consumption

**Fig 5:**
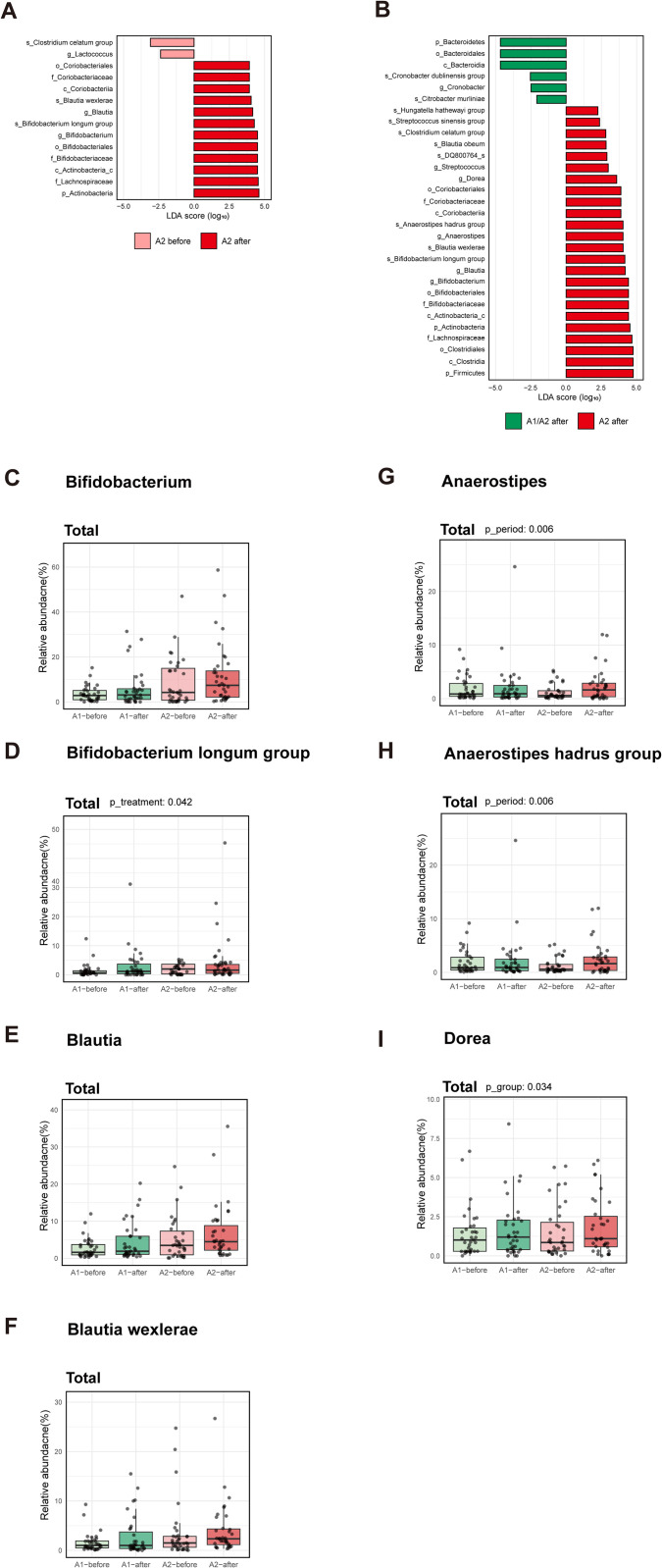
Identification of taxonomic biomarkers in the A2 milk group. (A-B) Linear discriminant analysis (LDA) effect size (LEfSe) analysis before and after consumption of A2 milk (A), and after consumption of A2 milk compared to A1/A2 milk (B). (C-I) Box plots for relative taxonomic abundance of genus *Bifidobacterium* (C), species *Bifidobacterium longum* (D), genus *Blautia* (E), species *Blautia wexlerae* (F), genus *Anaerostipes* (G), species *Anaerostipes hadrus* group (H), and genus *Dorea* (I). Statistical analysis for (C-I) was performed using a generalized linear model (GLM). Significant *p*-values for p_treatment (A2 treatment effect), p_period (period effect), and p_group (group effect) are displayed above the graphs.

LEfSe analysis was used to compute the differential abundance of taxa between the various groups. First, LEfSe analysis identified several taxa with significant changes in relative abundance before and after consumption of A2 milk (Fig 5A). Taxa enriched in the “before consumption of A2 milk” condition included the genus *Lactococcus* and the species *Clostridium celatum*. Taxa enriched in the “after consumption of A2 milk” condition included class Actinobacteria and Coriobacteria, order Bifidobacteriales and Coriobacteriales, family Lachnospiraceae, Bifidobacteriaceae, and Coriobacteriaceae, genus *Bifidobacterium* and *Blautia*, and species *Bifidobacterium longum* and *Blautia wexlerae*. For the comparison between “A1/A2 after” and “A2 after” conditions, the following taxa were significantly enriched (Fig 5B). In “A1/A2 after”: phylum Bacteroidetes, class Bacteroidia, order Bacteroidales, genus *Cronobacter*, and species *Cronobacter dublinensis* and *Cronobacter muytjensii*. In “A2 after”: phylum Firmicutes, class Clostridia, Actinobacteria, and Coribacteria, order Clostridiales, Bifidobacteriales, and Coriobacteriales, family Lachnospiraceae, Bifidobacteriaceae, and Coriobacteriaceae, genus *Bifidobacterium*, *Blautia*, *Anaerostipes*, *Dorea*, and *Streptococcus*, and species *Bifidobacterium longum*, *Blautia wexlerae*, *Anaerostipes hadrus*, DQ800764, *Blautia obeum*, *Clostridium celatum*, *Streptococcus sinensis*, and *Hungatella hathewayi*.

Detailed boxplots illustrate the relative abundance of specific taxa at the genus and species level across the various milk consumption conditions: The relative abundance of genus *Bifidobacterium* was significantly higher in the “A2 after” group compared with the “A2 before” (*p *= 0.001) and “A1/A2 after” (*p* = 0.015) groups (Fig 5C). No significant difference was observed between “A1/A2 before” and “A1/A2 after”, and between “A1/A2 before” and “A2 before” (Fig 5C). The species *Bifidobacterium longum* showed a significant increase in the “A2 after” compared with the “A2 before” (*p *= 0.006) and “A1/A2 after” (*p *= 0.023) groups (Fig 5D). No significant difference was observed between the “A1/A2 before” and “A1/A2 after” groups, and between the “A1/A2 before” and “A2 before” groups (Fig 5D). The genus *Blautia* was significantly more abundant in the “A2 after” group compared with the “A2 before” (*p *= 0.037) and “A1/A2 after” (*p *= 0.017) groups (Fig 5E). No significant differences were noted among the other comparisons (Fig 5E). A significant increase in species *Blautia wexlerae* was observed in the “A2 after” compared with the “A2 before” (*p* = 0.039) and “A1/A2 after” (*p* = 0.027) groups (Fig 5F). The genus *Anaerostipes*, the species *Anaerostipes hadrus*, and the genus *Dorea* were enriched in the “A2 after” group compared with the “A1/A2 after” group (*p *= 0.020, *p *= 0.022, and *p *= 0.047, respectively), and showed no statistical significance in the other comparison groups (Fig 5G–5I). Additionally, we performed a subgroup analysis by dividing the Total group into PA and AP (S4 Fig). The effect of A2 treatment was significant only in the PA group for *Anaerostipes* and the *Anaerostipes hadrus* group (S4E and S4F Fig; p_treatment = 0.002). Our LEfSe analysis highlighted significant shifts in the gut microbiome composition after milk consumption, particularly within the A2 group. The enrichment of beneficial species such as *Bifidobacterium longum* and *Blautia wexlerae* in the “A2 after” condition suggests a positive microbial response to the dietary intervention. These changes may be associated with improved gut health and potentially beneficial clinical outcomes.

### Identification of functional biomarkers after A2 milk consumption

**Fig 6:**
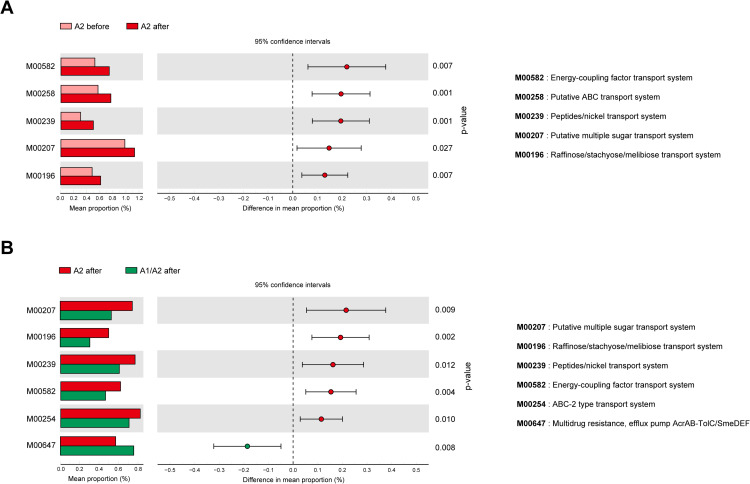
Identification of functional biomarkers in the A2 milk group. (A-B) Phylogenetic Investigation of Communities by Reconstruction of Unobserved States (PICRUSt) analysis before and after consumption of A2 milk (A), and after consumption of A2 milk compared to A1/A2 milk (B). The p-values were calculated for comparison difference between independent two groups.

To identify the functional composition and significant functional differences in microbiota between various groups, PICRUSt analysis was performed in conjunction with Dunnett’s test. PICRUSt analysis indicated significant differences in several KEGG modules in “before and after consumption of A2 milk” (Fig 6A). The KEGG modules significantly enriched in the “after consumption of A2 milk” group included: M00582 (Energy-coupling factor transport system), M00258 (Putative ATP‐binding cassette (ABC) transport system), M00239 (Peptides/nickel transport system), M00207 (Putative multiple sugar transport system), and M00196 (Raffinose/starchyose/melibiose transport system) (Fig 6A).

Similarly, comparing the “After consumption of A2 milk” to “A1/A2 milk” revealed significant differences in several KEGG modules (Fig 6B). Specifically, in the “A2 after” group, the enriched KEGG modules included: M00582 (Energy-coupling factor transport system), M00254 (ABC-2 type transport system), M00239 (Peptides/nickel transport system), M00207 (Putative multiple sugar transport system), and M00196 (Raffinose/starchyose/melibiose transport system) (Fig 6B). Additionally, in the “A1/A2 after” group, the KEGG module M00647 (Multidrug resistance, efflux pump AcrAB-TolC/SmeDEF) was found to be significantly enriched (Fig 6B).

### Spearman correlation analysis between taxonomic and functional biomarkers

**Fig 7:**
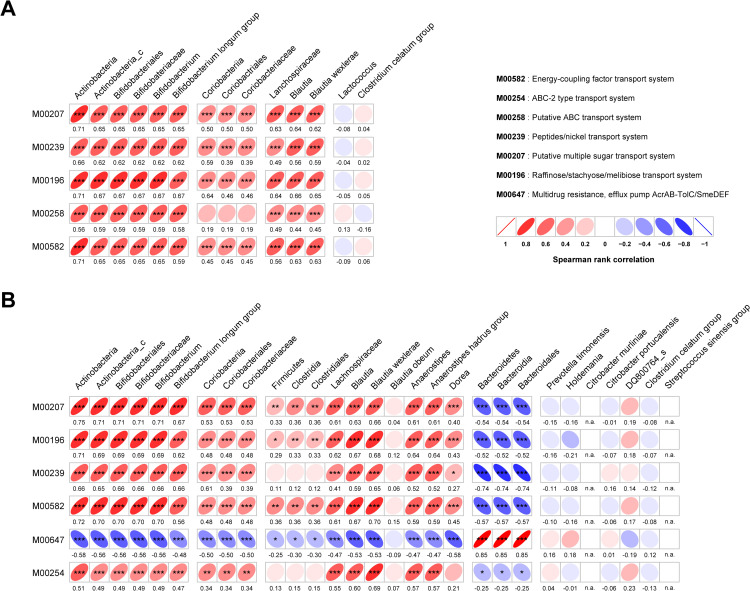
Relationships and predicted functions of gut microbiota. (A-B) Spearman’s correlation analysis between significant taxonomic and functional biomarkers before and after consumption of A2 milk (A), and after A2 milk consumption compared to A1/A2 milk consumption (B). Spearman’s rho values are indicated beneath each graph box. n.a., not assigned. The *p*-values were calculated for comparison difference between independent two groups. *, *p* < 0.05; **, *p* < 0.01; ***, *p* < 0.001.

Spearman’s correlation analysis was performed to explore the relationships between significantly enriched gut microbial taxa and functional KEGG modules. Before and after consumption of A2 milk, the phylogenies of *Bifidobacterium longum* and *Blautia wexlerae* exhibited significant positive correlations with enriched KEGG module transport systems (Fig 7A). The phylogeny of *Coriobacteriaceae* also showed significant positive correlations with all predicted KEGG module transport systems, though these correlations were lower compared to those of *B. longum* and *B. wexlerae* (Fig 7A). In contrast, *Lactococcus* and the *Clostridium celatum* group did not exhibit significant correlations with the predicted KEGG modules (Fig 7A).

After the consumption of either A1/A2 or A2 milk, *B. longum* and *B. wexlerae* continued to show positive correlations with enriched KEGG module transport systems (Fig 7B). The *Coriobacteriaceae* family followed a similar trend, though with lower correlations compared to *B. longum* and *B. wexlerae* (Fig 7B). Additionally, the *Anaerostipes hadrus* group and *Dorea*, both associated with butyrate production [30,31], were positively correlated with transport systems (Fig 7B). Conversely, *Bacteroidales* order exhibited a strong positive correlation with the multidrug resistance efflux pump AcrAB-TolC/SmeDEF (M00647), indicating an opposite trend compared to other taxa, such as *B. longum* and *B. wexlerae*, which were positively correlated with beneficial transport systems (Fig 7B). *Prevotella timonensis*, *Holdemania*, *Citrobacter portucalensis*, DQ800764_s, *Clostridium celatum* group, and *Streptococcus sinensis* group did not show significant correlations with the predicted KEGG modules (Fig 7B).

These results suggest that well-known beneficial microbes, such as *B. longum* and *B. wexlerae*, are strongly associated with energy, sugar, and peptide/nickel transport systems, supporting the distinct beneficial effects of consuming A2 milk. Additionally, *Coriobacteriaceae*, *Anaerostipes hadrus* group, and *Dorea* were associated with the same modules, highlighting the potential benefits of gut microbiome-based interventions through A2 milk consumption.

### Spearman correlation analysis between calprotectin and gut microbiota

**Fig 8:**
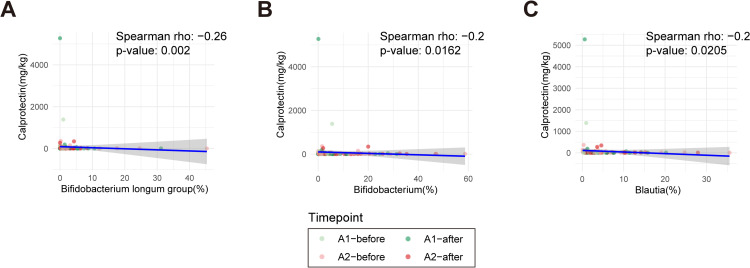
Correlation between calprotectin and gut microbiota. (A-C) Spearman’s correlation analysis between Calprotectin and *Bifidobacterium longum* group (A), *Bifidobacterium* genus (B), and *Blautia* genus (C). Spearman’s rho values and *p*-values are displayed above the graphs.

Furthermore, we analyzed the correlation between taxa identified as significant in the LEfSe analysis of pre- and post-intake data for A1 and A2 milk (Total, n = 35) and calprotectin levels, which showed significant changes after A2 intake in a previous study [27]. As a result, calprotectin showed a significant negative correlation with the *Bifidobacterium longum* group (Fig 8A, Spearman’s rho = -0.26, *p* = 0.002), *Bifidobacterium* genus (Fig 8B, Spearman’s rho = -0.20, *p* = 0.0162), and *Blautia* genus (Fig 8C, Spearman’s rho = -0.20, *p* = 0.0205). Additionally, we performed subgroup analysis by dividing the Total group into PA and AP. In the PA group, *Bifidobacterium longum* group and *Bifidobacterium* genus showed a significant negative correlation with calprotectin (S5A Fig; Spearman’s rho = -0.25 and -0.24, *p* = 0.0345 and 0.0439, respectively), following a trend similar to that observed in the Total group. In the AP group, a significant negative correlation was also found between *Bifidobacterium longum* group and calprotectin (S5A Fig; Spearman’s rho = -0.25, *p* = 0.0421). Taken together, these results suggest that *Bifidobacterium longum* group and *Bifidobacterium* genus may play a protective role against intestinal inflammation by negatively correlating with calprotectin levels.

## Discussion

In this randomized, crossover study, we aimed to evaluate the effects of A2 milk consumption on gut microbial communities in South Koreans who experience GI discomfort following milk consumption. Our results demonstrated that consuming A2 milk induced significant shifts in the gut microbial communities such as a marked enrichment of Actinobacteria and Firmicutes, particularly *Bifidobacterium longum* and *Blautia wexlerae*. These microbes were associated with KEGG module transport systems related to energy and nutrient processing in the gut. In addition, our beta diversity analysis revealed no significant changes in gut microbiota composition before and after the consumption of A1/A2 milk. However, the consumption of A2 milk resulted in a notable shift in gut microbiota composition, indicating that A2 milk has a unique effect on the gut microbiome. This significant alteration of the microbial community of the gut suggests that A2 milk consumption may promote gut bacterial taxa associated with beneficial health outcomes [27].

Our present findings align with previous studies that have demonstrated the influence of diet on gut microbiota composition [22,32]. Specifically, our research shows that while both A1/A2 milk and A2 milk influence the composition of gut microbiota, they do not significantly affect the overall diversity of the gut microbiome. In terms of alpha diversity, our analysis indicated no substantial changes across the various experimental groups and conditions. While stability in alpha diversity is often associated with a resilient gut microbiota, it does not necessarily preclude alterations in microbial composition or function. Notably, shifts in microbial community structure and metabolic activity can occur independently of alpha diversity changes [33], and certain dietary interventions, such as increased fiber intake, have been shown to reduce alpha diversity while simultaneously enhancing gut health through mechanisms such as increased short-chain fatty acid (SCFA) production [34]. Given these considerations, our findings suggest that the consumption of A1/A2 milk, with its 40:60 ratio of A1 to A2 beta-casein, and A2 milk, made entirely of A2 beta casein, does not induce pronounced disruptions in gut microbial diversity. However, it is important to recognize that alpha diversity alone is an insufficient marker for dysbiosis. A more comprehensive assessment incorporating microbial compositional and functional analyses would be necessary to fully elucidate the impact of these milk types on gut microbiome stability and host health [35].

Our taxonomic analysis revealed significant changes in the relative abundance of specific gut microbial taxa following the consumption of A2 milk. Notably, there was a significant increase in Actinobacteria and Firmicutes, particularly *Bifidobacterium* and *Blautia*, after A2 milk consumption. *Bifidobacterium* is a well-known beneficial gut microbe associated with numerous health benefits, including improved digestion and enhanced immune function [36]. The increase in *Bifidobacterium longum* species abundance post-A2 milk consumption suggests a potential mechanism for the alleviation of GI discomfort [37], as these microbes are known to produce short-chain fatty acids that support gut health [38,39]. We have also reported previously that *B. longum* reduced repeated water avoidance stress-sinduced gut dysbiosis in Wistar rats particularly in females, by modulating gut microbiota composition and reducing mast cell infiltration [40]. Similarly, we have shown that *Roseburia faecis* alleviates stress-induced irritable bowel syndrome in Wistar rats by decreasing fecal pellet output, modulating gut microbiota composition, and reducing mast cell infiltration, with notable effects in both males and females [41]. In the present study, we observed a significant increase in *Blautia* genus and *Blautia wexlerae* species after A2 milk consumption, suggesting a potential gut-modulating effect of A2 milk. *Blautia wexlerae* is known to have anti-inflammatory properties and is associated with a healthy gut microbiome [42]. The microbial changes observed exclusively after A2 milk consumption, but not after A1/A2 milk intake, underscore the distinct influence of A2 milk on gut microbiota composition. While our study does not directly investigate stress-induced dysbiosis, the observed microbial shifts align with previous research indicating that dietary interventions can promote gut microbiota stability, potentially enhancing resilience against various physiological and dietary stressors.

Furthermore, the interaction between A1 β-casein and the release of BCM-7 has been linked to delayed gastric emptying and increased GI transit time, potentially exacerbating symptoms of lactose intolerance [5]. Our findings support the hypothesis that consuming A2 milk, which does not produce BCM-7, may reduce GI discomfort by promoting a healthier gut microbiota composition and enhancing gut motility. Given the high prevalence of self-reported lactose intolerance in South Korea, our study provides important insights into the potential benefits of A2 milk consumption as an alternative to drinking regular A1/A2 milk. The significant shifts in beneficial gut microbes and the absence of BCM-7-related effects suggest that A2 milk consumption may offer a dietary solution for individuals with lactose intolerance or milk-related GI discomfort [12].

The functional analysis using PICRUSt revealed that after A2 milk consumption, compared to before A2 milk consumption or after A1/A2 milk consumption, several KEGG modules related to transport systems, including those involved in energy-coupling factor, ABC, multiple sugars, raffinose/starchyose/melibiose, and peptide/nickel transport, were commonly enriched. These findings highlight potential metabolic and physiological impacts of A2 milk on gut microbiota functionality. ECF transport systems enhance bacterial vitamin uptake, promoting gut resilience and nutrient absorption [43], while ABC transporters facilitate the transport of diverse substrates, potentially improving digestion, immune modulation, and intestinal barrier function [44]. Increased activity in sugar and oligosaccharide transport systems supports carbohydrate metabolism, SCFA production, and microbial diversity, benefiting conditions like lactose intolerance and IBS [45,46]. Furthermore, peptide transport systems play a crucial role in nitrogen metabolism and bacterial growth [47], while nickel transporters support enzymatic functions involving hydrogenases and ureases [48]. Their enrichment suggests enhanced microbial nitrogen utilization and metal cofactor metabolism, contributing to gut microbiota stability, reduced gastrointestinal infections, and improved metabolic health [47,48]. These findings collectively highlight the potential of A2 milk which might support gut bacterial taxa associated with beneficial health outcomes. The positive correlation between these functional modules and the enriched taxa, such as *Bifidobacterium longum* and *Blautia wexlerae*, further supports the notion that A2 milk promotes beneficial metabolic functions in the gut microbiota. In contrast, the strong correlation between the *Bacteroidales* order in the A1/A2 after group and the multidrug resistance efflux pump (M00647) suggests potential microbial functional adaptations rather than necessarily indicating an increase in antimicrobial resistance. Multidrug resistance efflux pumps play diverse roles in bacterial physiology beyond resistance mechanisms, including environmental adaptation and interspecies competition [49]. While these functional changes may influence microbial composition and metabolic activities, including carbohydrate metabolism and SCFA production, the implications for gut health remain speculative [50,51]. Further research is needed to determine whether such shifts are transient or indicative of broader microbial community restructuring. While prior studies have suggested that A1 beta-casein might contribute to adverse gastrointestinal outcomes and inflammation [52], the current findings should be interpreted with caution, as correlation does not imply causation.

In this study, we found a significant negative correlation between fecal calprotectin levels and both the *Bifidobacterium* genus and the *B. longum* group, suggesting a potential protective role of these bacteria against intestinal inflammation. These findings align with previous studies indicating that A1 β-casein may contribute to increased intestinal inflammation, while A2 β-casein has been associated with a lower inflammatory response [12,52]. Given the critical role of gut microbiota in regulating immune responses and maintaining intestinal health, our results suggest that dietary interventions targeting microbiota composition may help mitigate inflammation. However, further large-scale studies incorporating clinical symptoms, microbiota profiling, and inflammatory markers are necessary to confirm these associations and elucidate underlying mechanisms.

There are several limitations in our study. The sample size of this study was relatively small, and the study population was limited to individuals who reported GI discomfort after milk consumption. Future studies need to include larger, more diverse populations to validate these findings and explore the long-term effects of A2 milk consumption on gut health. Additionally, while we observed significant changes in the abundance of specific gut microbial taxa, further research is needed to elucidate the mechanisms underlying these shifts and their implications for overall health. Metagenomics and metabolomics analyses may provide deeper insights into the functional changes in the gut microbiome induced by A2 milk consumption [53]. Moreover, our study utilized “metataxonomics”, which involves sequencing a specific marker region—in this case, the 16S rRNA gene. While this method allowed for faster data analysis based on the known bacterial genome, it lacks precision. Furthermore, although this study focused on microbiota composition, including predicted functionality using PICRUSt, it still lacks comprehensive functional information. The functional profiling we conducted was predictive, relying on the KEGG database. The “metagenomics” with whole-genome shotgun sequencing might have provided insight for underlying metabolic changes. However, due to resource limitations, it was not feasible to perform whole-genome sequencing.

In conclusion, our study demonstrates that A2 milk consumption significantly alters gut microbiota composition in individuals experiencing GI discomfort after milk consumption. This dietary intervention promotes the abundance of beneficial microbes such as from the genus *Bifidobacterium* and *Blautia*, which show a negative correlation with calprotectin levels, suggesting a potential protective role against intestinal inflammation. Our findings suggest that A2 milk consumption may serve as a beneficial dietary alternative for those with lactose intolerance or milk-related GI discomfort, potentially improving gut health and alleviating GI discomfort, potentially improving gut health.

## Supporting information

S1 Table Eligibility criteria. (DOCX)

S1 Fig Beta diversity of gut microbiota in the PA and AP groups. Sample clustering by Generalized UniFrac-based PCoA at the species level. (A) PCoA plot comparing A2 milk and A1/A2 milk before consumption in the PA and AP groups. (B) PCoA plot comparing samples before and after A1/A2 milk consumption in the PA and AP groups. (C) PCoA plot comparing A2 milk and A1/A2 milk after consumption in the PA and AP groups. (D) PCoA plot comparing samples before and after A2 milk consumption in the PA and AP groups. Significance for similarity of bacterial population clustering was analyzed by PERMANOVA. *, *p* < 0.05, N.S., no significance. The clustering of each group is marked with a different color: A1/A2 before, yellowish green ellipse; A1/A2 after, green ellipse; A2 before, pink ellipse; A2 after, red ellipse. PCoA, principal coordinates analysis; PERMANOVA, permutational multivariate analysis of variance.
(DOCX)

S2 Fig Alpha diversity of gut microbiota in the PA and AP groups. (A) ACE, (B) Chao1, (C) Jackknife, (D) Observed OTU count, (E) Shannon, (F) NPShannon, (G) Simpson, (H) Phylogenetic Diversity, (I) Good’s coverage of library.
(DOCX)

S3 Fig Distribution of gut microbiome before and after consumption of A1/A2 and A2 milk in the PA and AP groups. (A-C) Gut microbiota compositions at the phylum (A), family (B), and genus (C) levels in the PA and AP groups. (D-H) Box plots for relative taxonomic abundance of Firmicutes (D), Bacteroidetes (E), Actinobacteria (F), Verrucomicrobia (G), and Proteobacteria (H) in the PA and AP groups. Statistical analysis for (D-H) was performed using a generalized linear model (GLM). Significant *p*-values for p_treatment (A2 treatment effect), p_period (period effect), and p_group (group effect) are displayed above the graphs. Only p_treatment effects were significant, and these are indicated in the figures.
(DOCX)

S4 Fig Identification of taxonomic biomarkers in the PA and AP groups. Box plots for relative taxonomic abundance of genus *Bifidobacterium* (A), species *Bifidobacterium longum* (B), genus *Blautia* (C), species Blautia wexlerae (D), genus *Anaerostipes* (E), species *Anaerostipes hadrus* group (F), and genus *Dorea* (G) in the PA and AP groups. Statistical analysis was performed using a generalized linear model (GLM). Significant *p*-values for p_treatment (A2 treatment effect), p_period (period effect), and p_group (group effect) are displayed above the graphs. Only p_treatment effects were significant, and these are indicated in the figures.
(DOCX)

S5 Fig Correlation between calprotectin and gut microbiota in the PA and AP groups. Spearman’s correlation analysis between calprotectin and gut microbiota in the PA (A) and AP (B) groups. Spearman’s rho values and *p*-values are displayed above the graphs. (DOCX)
