# Supplementary material for: Beneficial effect of consuming milk containing only A2 beta-casein on gut microbiota: A single-center, randomized, double-blind, cross-over study
Source: PLoS One. 2025 May 8;20(5):e0323016. doi: 10.1371/journal.pone.0323016 (PMC12061139; doi:10.1371/journal.pone.0323016)
Supplement: S1 Table — (DOCX) [file pone.0323016.s001.docx]

**S1 Table. Eligibility criteria.**

| Selection criteria | - Individuals aged between 20 and 70 years old - Individuals who experience digestive discomfort related to regular milk consumption, such as bloating, gas, fullness, abdominal pain, rumbling, borborygmus, and urgency of bowel movements, as assessed in Visit 1 (the score for each symptom in the Gastrointestinal Symptom score must be 2 or lower) - Individuals who agree to participate in the study and sign the informed consent form |
| --- | --- |
| Exclusion Criteria | - Individuals currently undergoing treatment for severe cardiovascular, immune, respiratory, gastrointestinal/hepatobiliary, renal and urinary, neurological, musculoskeletal, psychiatric, infectious, metabolic diseases, or malignancies - Individuals with a history of gastrointestinal diseases, including irritable bowel syndrome, inflammatory bowel disease, or celiac disease, or those who have undergone gastrointestinal surgery - Individuals all symptom scores of 0 or at least one symptom score of 3 in the Gastrointestinal Symptom score - Individuals with severe lactose intolerance - Individuals with a history of fecal impaction - Individuals with alcohol addiction or drug abuse - Individuals who have been hospitalized within the past 3 months prior to Visit 1 - Individuals who have taken medications that affect body weight within 3 months prior to Visit 1, including anti-obesity drugs (appetite suppressants, fat absorption inhibitors, GLP-1 receptor agonists, etc.), psychiatric medications for depression or schizophrenia, diuretics, contraceptives, steroids, female hormone therapy, or thyroid hormone therapy - Individuals who have taken immunosuppressants or anti-inflammatory drugs within 30 days prior to Visit 1 - Individuals who have received antibiotics or bowel-cleansing agents within 2 weeks prior to Visit 1 - Individuals who have taken gastrointestinal motility enhancers (such as 5-HT4 agonists, D2 antagonists, or cholinergic agonists), laxatives (such as fiber supplements [psyllium, methylcellulose], stool softeners, osmotic laxatives [sorbitol, lactulose], stimulant laxatives [bisacodyl, anthraquinones]), or probiotics within 1 week prior to Visit 1 - Pregnant or breastfeeding women, or those planning to become pregnant during the study period - Individuals with dairy allergies - Individuals who have participated in another interventional clinical trial (including human application trials) within the past 3 months prior to Visit 1 or plan to participate in another interventional clinical trial during this study - Individuals deemed inappropriate for the study by the investigator |
