# Supplementary figures and images for: Beneficial effect of consuming milk containing only A2 beta-casein on gut microbiota: A single-center, randomized, double-blind, cross-over study
Source: PLoS One. 2025 May 8;20(5):e0323016. doi: 10.1371/journal.pone.0323016 (PMC12061139; doi:10.1371/journal.pone.0323016)

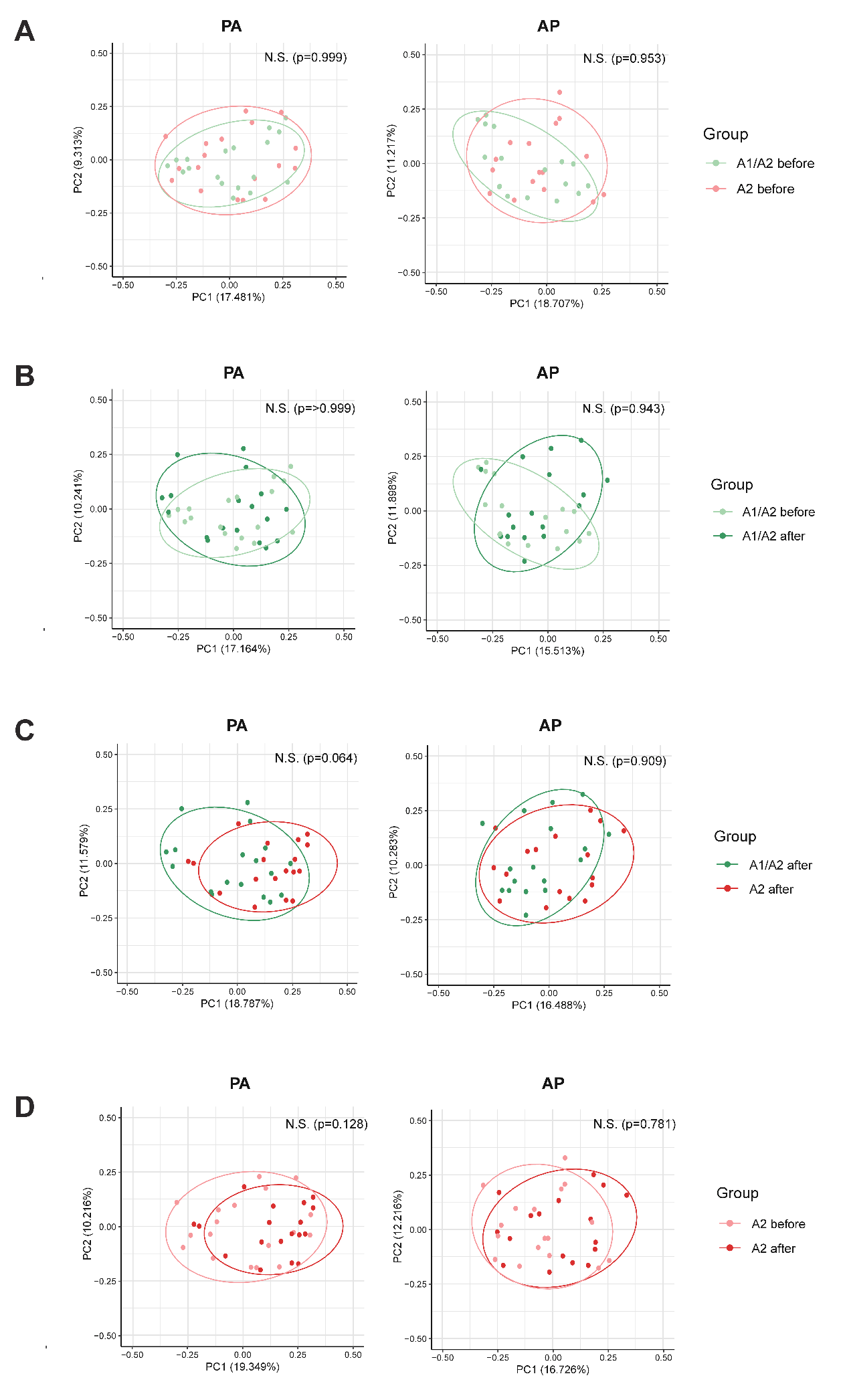

Supplement: S1 Fig — Sample clustering by Generalized UniFrac-based PCoA at the species level. (A) PCoA plot comparing A2 milk and A1/A2 milk before consumption in the PA and AP groups. (B) PCoA plot comparing samples before and after A1/A2 milk consumption in the PA and AP groups. (C) PCoA plot comparing A2 milk and A1/A2 milk after consumption in the PA and AP groups. (D) PCoA plot comparing samples before and after A2 milk consumption in the PA and AP groups. Significance for similarity of bacterial population clustering was analyzed by PERMANOVA. *, p < 0.05, N.S., no significance. The clustering of each group is marked with a different color: A1/A2 before, yellowish green ellipse; A1/A2 after, green ellipse; A2 before, pink ellipse; A2 after, red ellipse. PCoA, principal coordinates analysis; PERMANOVA, permutational multivariate analysis of variance. (DOCX) [file pone.0323016.s002.docx]

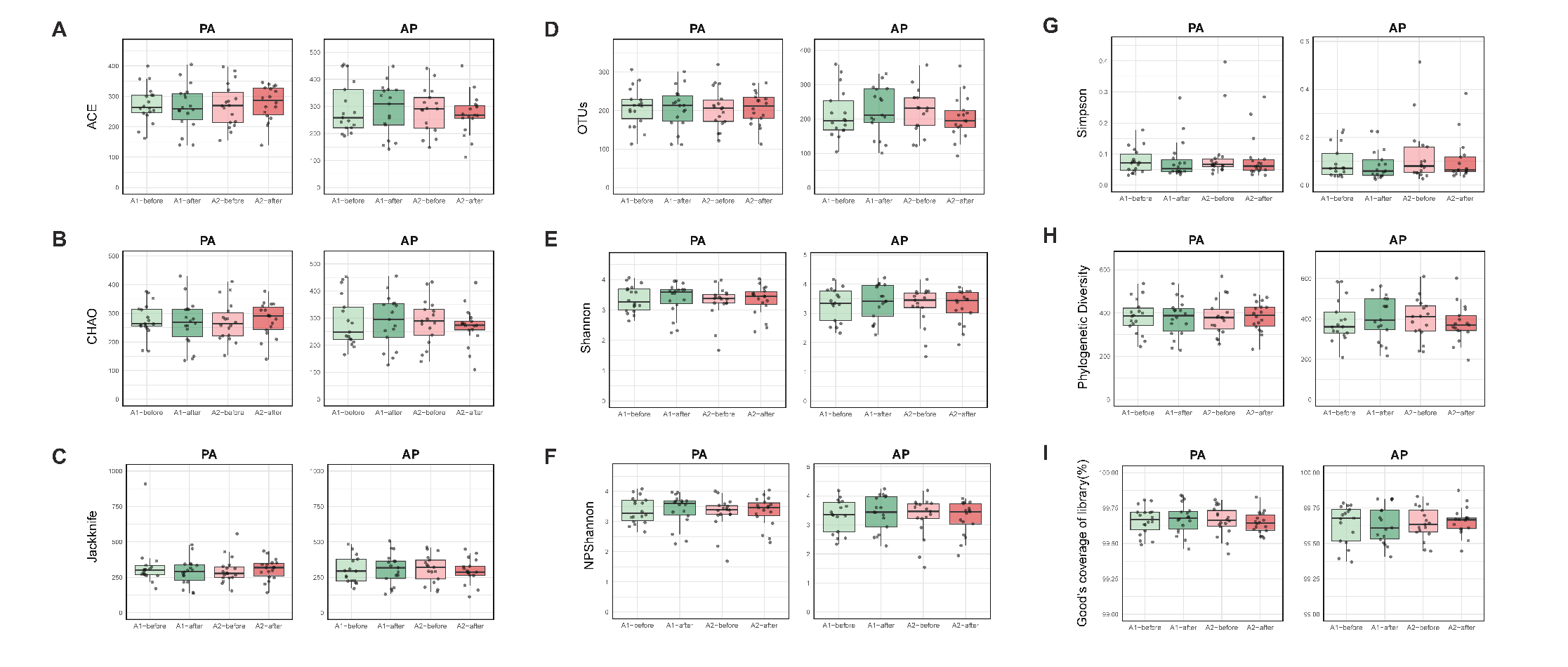

Supplement: S2 Fig — (A) ACE, (B) Chao1, (C) Jackknife, (D) Observed OTU count, (E) Shannon, (F) NPShannon, (G) Simpson, (H) Phylogenetic Diversity, (I) Good’s coverage of library. (DOCX) [file pone.0323016.s003.docx]

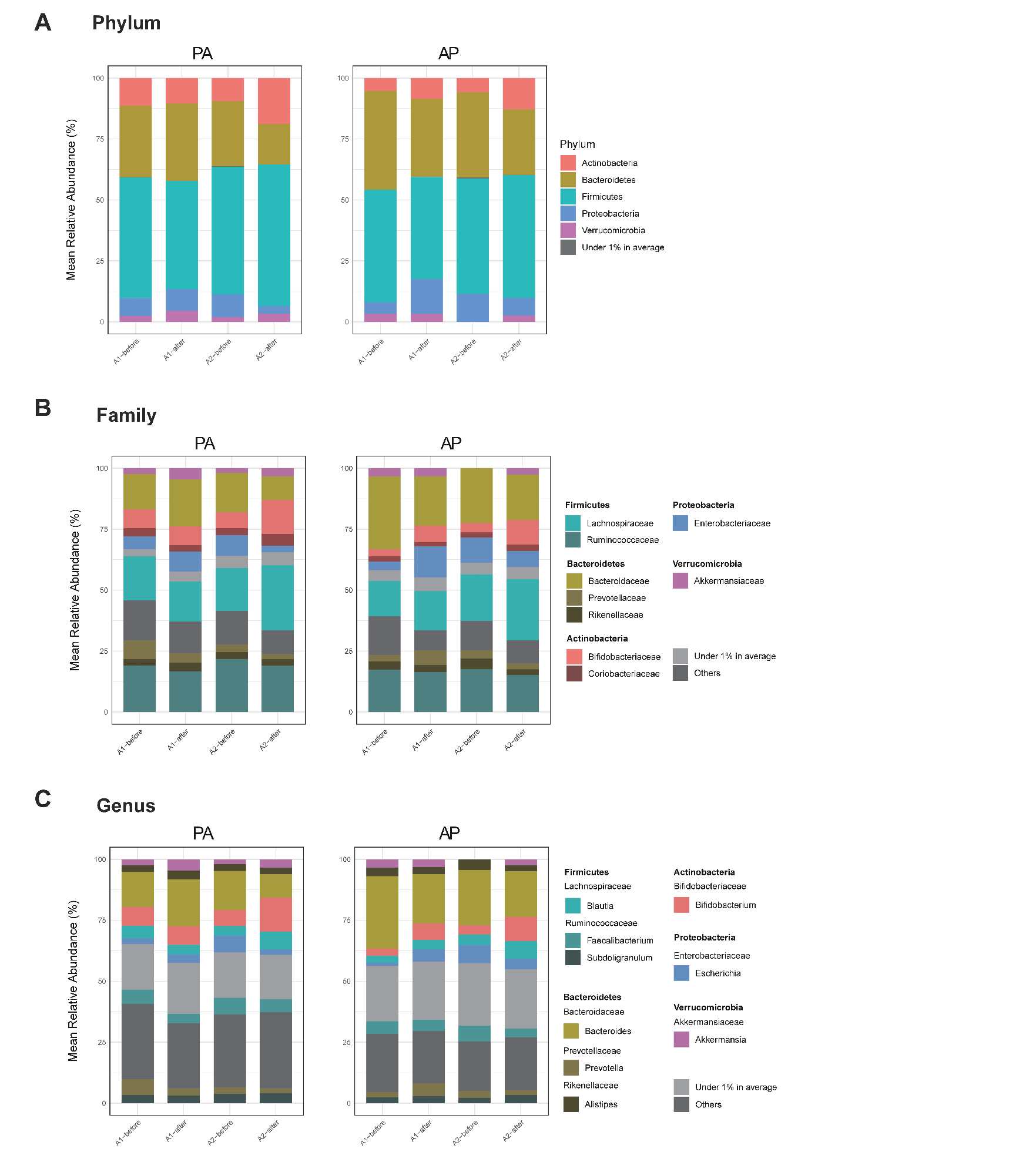


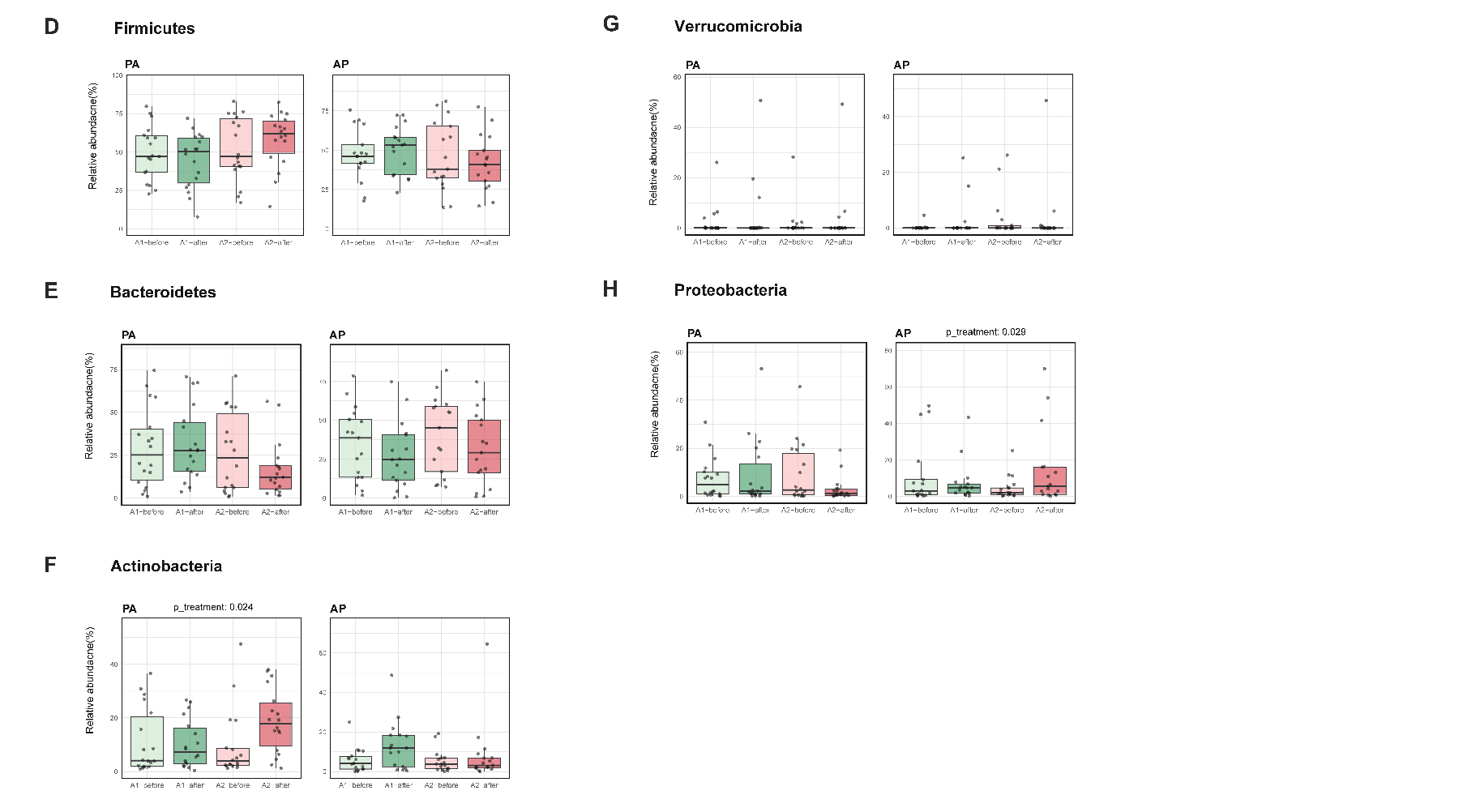

Supplement: S3 Fig — (A-C) Gut microbiota compositions at the phylum (A), family (B), and genus (C) levels in the PA and AP groups. (D-H) Box plots for relative taxonomic abundance of Firmicutes (D), Bacteroidetes (E), Actinobacteria (F), Verrucomicrobia (G), and Proteobacteria (H) in the PA and AP groups. Statistical analysis for (D-H) was performed using a generalized linear model (GLM). Significant p-values for p_treatment (A2 treatment effect), p_period (period effect), and p_group (group effect) are displayed above the graphs. Only p_treatment effects were significant, and these are indicated in the figures. (DOCX) [file pone.0323016.s004.docx]

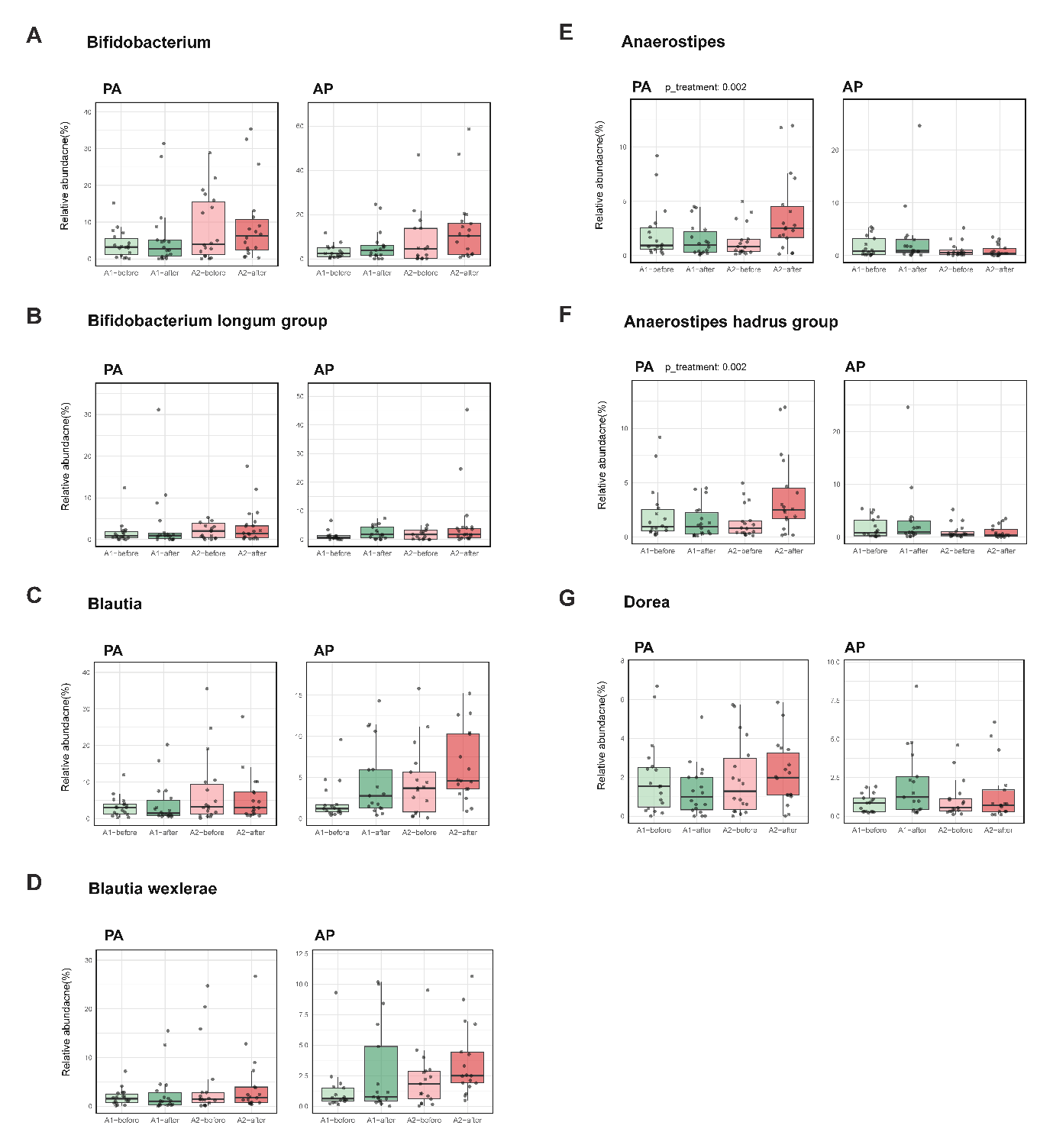

Supplement: S4 Fig — Box plots for relative taxonomic abundance of genus Bifidobacterium (A), species Bifidobacterium longum (B), genus Blautia (C), species Blautia wexlerae (D), genus Anaerostipes (E), species Anaerostipes hadrus group (F), and genus Dorea (G) in the PA and AP groups. Statistical analysis was performed using a generalized linear model (GLM). Significant p-values for p_treatment (A2 treatment effect), p_period (period effect), and p_group (group effect) are displayed above the graphs. Only p_treatment effects were significant, and these are indicated in the figures. (DOCX) [file pone.0323016.s005.docx]

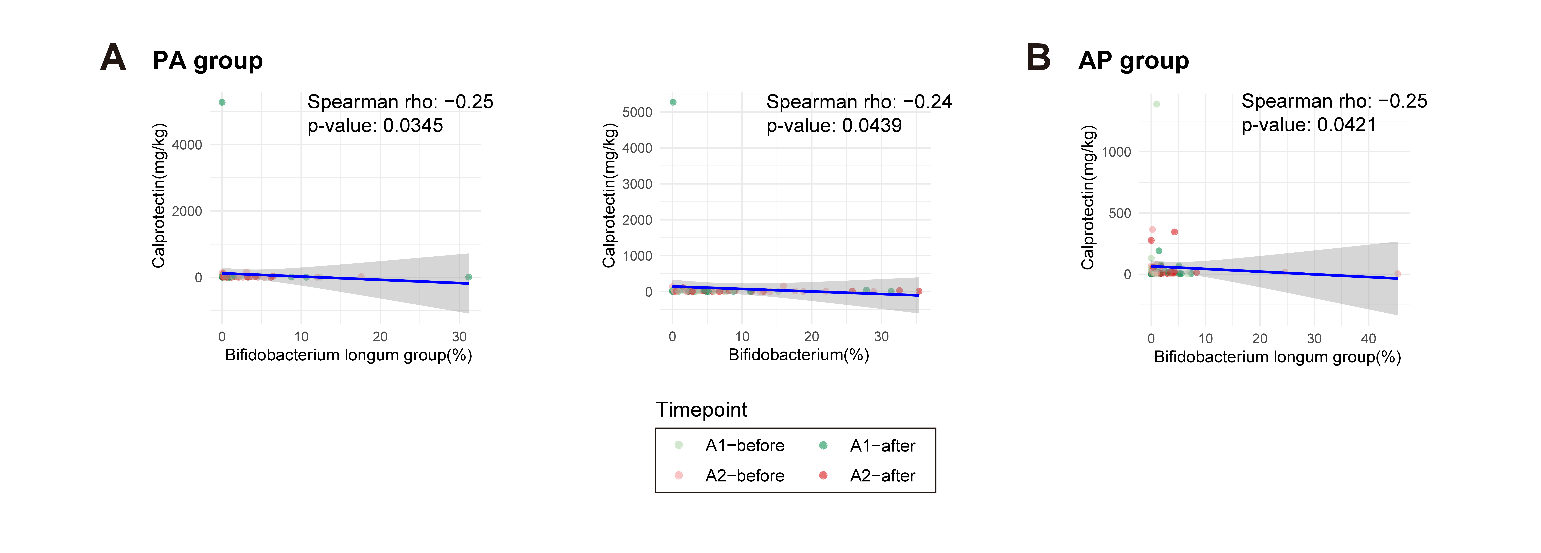

Supplement: S5 Fig — (DOCX) [file pone.0323016.s006.docx]
